# Intrinsic mechanical vibrations as a missing dimension in amyloid-*β* clearance: a mechanochemical hypothesis for Alzheimer’s disease

**DOI:** 10.3389/fnagi.2026.1749562

**Published:** 2026-01-20

**Authors:** Xiaochen Lai

**Affiliations:** School of Future Technology, Nanjing University of Information Science & Technology, Nanjing, China

**Keywords:** Alzheimer’s disease, amyloid-*β*, BBB transporter, snoring, vibration, vocalization

## Abstract

Alzheimer’s disease is widely viewed as a disorder of disturbed amyloid-*β* (Aβ) homeostasis, yet it remains unclear why Aβ shifts from routine turnover to progressive retention and plaque growth in mid-life. This article advances a mechanochemical hypothesis: that age-related reductions in low-intensity mechanical vibrations in the head and neck—arising from everyday self-vocalization (speech, singing, humming) and physiological, non-hypoxic upper-airway motion during stable sleep—contribute to a gradual narrowing of the Aβ clearance bottleneck. Gentle vibrations transmitted through bone and soft tissue could, in principle, enhance interstitial mixing, reduce local supersaturation and nucleation, increase encounter rates at blood–brain barrier and meningeal-lymphatic interfaces, and augment perivascular and glymphatic transport. A sustained decline in this “intrinsic mechanical vibration” (IMV) dose in mid-life might therefore favor Aβ retention without any change in production. We bring together indirect lines of evidence that are compatible with this view, including sleep and glymphatic studies, neuromodulatory stimulation paradigms, music and singing interventions, and hearing-loss epidemiology, and we discuss domains that at first appear inconsistent, such as voice-heavy occupations and obstructive sleep apnea. We then outline a testable cascade that links behavioral and sleep changes in mid-life to reduced mechanical drive and impaired clearance, and we propose concrete experiments spanning human laboratory studies, clinical trials, animal models, and prospective cohorts. Whether ultimately supported or refuted, this IMV–clearance framework highlights mechanical forces as a potentially modifiable dimension of Alzheimer’s disease risk and treatment response, alongside sleep, vascular health, and cognitive engagement.

## 1 Introduction

Alzheimer’s disease (AD) is the most common neurodegenerative disorder and an increasing burden for patients, caregivers, and health systems worldwide (GBD 2019 Dementia Forecasting Collaborators, 2022). With population aging, AD prevalence and associated costs are projected to rise sharply in coming decades. Despite substantial progress in understanding its biology and developing treatments, two questions remain only partially answered: why Aβ deposition accelerates in mid-life, and why clearance mechanisms that are adequate earlier in life begin to fail later on, even when production remains comparatively stable.

Several major frameworks have shaped thinking about AD. The amyloid cascade hypothesis emphasizes Aβ aggregation and toxicity, typically framed as an imbalance between production and clearance (Selkoe and Hardy, 2016). The tau hypothesis focuses on abnormal phosphorylation and propagation of tau, with resulting tangles and circuit disruption (Bloom, 2014). Neuroinflammatory (Chen and Yu, 2023), vascular (Zlokovic, 2011), metabolic (de la Monte, 2014), and cholinergic (Breijyeh and Karaman, 2020) perspectives each add important detail about glia, blood flow, energetics, and neurotransmission. Yet across these viewpoints, a consistent puzzle remains (Tarasoff-Conway et al., 2015; Mawuenyega et al., 2010): what changes in the physical or behavioral environment of the brain make it easier for Aβ to accumulate and harder for it to be cleared, often starting in mid-life?

Recent work has shifted more attention to clearance. Glymphatic (Iliff et al., 2012) and meningeal-lymphatic pathways (Louveau et al., 2015), arterial pulsatility (Mestre et al., 2018), sleep architecture and slow-wave–CSF coupling (Fultz et al., 2019), and receptor-mediated transport at the blood–brain barrier (BBB) (Zlokovic, 2011; Deane et al., 2003) all highlight the importance of fluid flow and solute transport. These systems are not purely biochemical; they are driven by pressure oscillations, tissue motion, and shear forces. This suggests the possibility that other mechanical inputs could matter as well.

Here we focus on one such candidate: low-frequency mechanical vibrations that arise from everyday vocalization and benign upper-airway motion and that propagate through the head and neck. We refer to these as intrinsic mechanical vibrations (IMVs). In humans, talking, reading aloud, singing, humming, and quiet, non-hypoxic snoring during stable sleep all generate vibration in the larynx, pharynx, and cranial structures. These vibrations are transmitted intracranially via bone and soft tissues and could influence interstitial mixing, perivascular and glymphatic flow, and interaction with clearance interfaces.

Day-to-day vocal activity and sleep duration may decline in mid-life. Time-use analyses and longitudinal panel studies indicate that everyday social exposure tends to contract with age: people spend less time “with others” in daily life, and personal social networks and contact frequency often shrink across adulthood (Marcum, 2013; Kalmijn, 2012). Although these data do not quantify vocal output directly, fewer and shorter social interactions plausibly translate into fewer opportunities for spontaneous conversation and casual self-vocalization. In parallel, objective sleep studies show that sleep often becomes shorter and/or more fragmented with age (Ohayon et al., 2004; Mander et al., 2017; Van Cauter et al., 2000), reducing time spent in consolidated, non-hypoxic sleep that may include benign upper-airway vibration. Together, these behavioral and sleep changes could lower cumulative exposure to such benign IMVs without requiring any change in Aβ production. In this hypothesis paper, we explore the idea that a sustained decline in this simple, mechanical resource contributes to Aβ clearance failure. We (i) define a conceptual framework for IMVs as a modulator of Aβ clearance, (ii) summarize evidence that points in the expected direction or seems to contradict it, (iii) propose concrete, falsifiable predictions and experimental strategies, and (iv) discuss how IMV-related interventions might one day complement disease-modifying Aβ therapies and other emerging approaches. The aim is not to replace existing models, but to add a mechanistically grounded dimension that can be tested with available tools.

## 2 Conceptual framework: intrinsic mechanical vibrations and Aβ clearance

### 2.1 Sources and definition of IMVs

For conceptual purposes, it is useful to think of IMV exposure as a measurable “dose” characterized by (i) intensity, (ii) duration, and (iii) spectral content over time. Practically, we suggest a two-tier dosimetry approach. First, source-proximal monitoring can quantify vocal-fold/upper-airway vibration using a neck-surface accelerometer (or contact microphone) that has already been validated for ambulatory vocal monitoring (Mehta et al., 2012; Lien and Stepp, 2014; Lei et al., 2022). Second, cranial transmission can be quantified using lightweight tri-axial accelerometers placed at reproducible skull locations (e.g., mastoid/temporal bone and forehead/vertex) to capture band-limited RMS acceleration (a_RMS) and/or a vibration dose value (VDV-style cumulative metric) integrated over time within pre-specified bands (e.g., 20–200 Hz and 200–500 Hz). This enables an explicit dose–response analysis and allows matched-dose experimental controls (e.g., external vibro-stimulation calibrated to the same skull acceleration as self-vocalization). Importantly, aging could reduce this cumulative dose through behavioral and sleep changes even if Aβ production remains stable.

### 2.2 Operationalizing IMV dose and defining a safe testing window

At present, a definitive “threshold” for IMV-supported clearance is unknown, and identifying an optimal window is itself a central test of the hypothesis. A conservative starting point is to define naturalistic IMV ranges using ambulatory dosimetry in healthy adults (e.g., typical daily speaking/singing/humming and stable, non-hypoxic sleep) and treat these as an initial safety-anchored envelope. Early mechanistic studies can then probe dose–response by varying duration and spectral emphasis within this physiological envelope while continuously monitoring symptoms and sleep quality.

A key design principle is to separate mechanical dose from cognitive/affective dose. This can be achieved by calibrating external vibro-stimulation to match measured skull acceleration during self-vocalization, and by using active controls that match attention and arousal without adding vocal vibration (e.g., silent reading, paced breathing, or listening tasks). A dose window would be considered “promising” only if biomarker changes track the measured mechanical dose after accounting for sleep architecture, hypoxia indices, and arousal.

### 2.3 Mechanistic routes from IMVs to clearance

We propose three main routes by which IMVs could support Aβ clearance:

1. Enhanced interstitial mixing and anti-nucleation effects: In liquids, gentle oscillatory motion can accelerate effective diffusion and mixing (e.g., via Taylor–Aris dispersion and related phenomena), helping to keep solutes more evenly distributed and may discourage local supersaturation and nucleation (Ottino and Wiggins, 2004; Suh and Kang, 2010; Frommelt et al., 2008). By analogy, small mechanical perturbations of interstitial fluid could reduce local Aβ hotspots and lower the probability of aggregation.
2. Increased encounter rates at clearance interfaces: Aβ efflux across the BBB and into meningeal-lymphatic channels depends on encounters between Aβ molecules and transport interfaces (such as LRP1 on endothelial cells or perivascular exit routes). Superimposed low-frequency displacements may increase the effective collision rate between Aβ and these interfaces, slightly increasing efflux per unit time even if transporter expression does not change.
3. Augmented perivascular and glymphatic pumping: Glymphatic and meningeal-lymphatic flows are driven in part by arterial pulsatility, respiration, and other mechanical inputs (Mestre et al., 2018; Iliff et al., 2012; Dreha-Kulaczewski et al., 2015). Additional gentle vibrations could strengthen pressure gradients and shear along perivascular spaces, modestly enhancing convective transport and solute removal.

Taken together, these mechanisms suggest that a chronic decline in IMV dose could narrow the clearance bottleneck and tilt the balance toward net Aβ retention, even if production remains stable.

### 2.4 A mechanochemical cascade

The proposed cascade can be summarized as follows:

1. Mid-life behavioral and sleep changes—less speech, singing, and humming outside of work, and shorter or more fragmented sleep with reduced time in stable, non-hypoxic snoring stages.
2. Reduced mechanical drive—lower cumulative exposure of the head and neck to low-frequency vibration arising from vocalization and benign upper-airway motion.
3. Slightly weaker transport—small decreases in interstitial mixing, perivascular/glymphatic flux, and encounter rates at BBB and meningeal-lymphatic interfaces.
4. Aβ retention and nucleation—higher local Aβ supersaturation, more frequent nucleation events, and progressive plaque expansion, with downstream tau pathology and neurodegeneration.

This cascade is meant to describe a modulatory axis rather than a primary cause. IMVs are proposed to act alongside genetic risk, vascular and metabolic factors, and other established influences on production and clearance.

## 3 Evidence consistent with IMV-supported clearance

Before reviewing the literatures below, we want to be explicit about the evidentiary status of this hypothesis. At present, we do not have direct experimental evidence that everyday vocalization or benign upper-airway vibration increases brain-to-blood Aβ transport or improves net Aβ clearance. Moreover, nearly all of the “supporting” domains discussed here—sleep physiology, 40-Hz stimulation, ultrasound, music/singing interventions, and hearing-loss epidemiology—can be reasonably explained by established mechanisms (e.g., sleep architecture and arousal, neurovascular coupling, cognitive/affective engagement, respiratory physiology, or general health/behavioral confounding) without invoking IMVs.

Accordingly, we present this section not as cumulative proof, but as a “directional compatibility” check: these literatures often change in a direction that is not inconsistent with an IMV modifier, while remaining fully compatible with other explanations. The hypothesis should therefore be evaluated primarily by the discriminating, falsifiable experiments outlined later (Sections 5 and 6.3), which are designed to separate mechanical vibration from cognitive/social and physiological confounds.

### 3.1 Sleep, glymphatic function, and Aβ

Insufficient or fragmented sleep elevates dementia risk, and shorter mid-life sleep duration predicts higher incident dementia (Sabia et al., 2021). Acute sleep deprivation in humans elevates Aβ PET signal in hippocampus and thalamus and increases CSF Aβ species (Shokri-Kojori et al., 2018; Ooms et al., 2014; Lucey et al., 2018). Mechanistic work links consolidated slow-wave sleep to enhanced glymphatic flow and coupled slow-wave, hemodynamic, and CSF oscillations that promote solute clearance (Xie et al., 2013; Fultz et al., 2019).

These findings independently support a robust “sleep → clearance → Aβ” link. Within our framework, reduced sleep duration and altered sleep architecture may also reduce nocturnal exposure to benign cranio-cervical vibrations (a component of the overall IMV dose) and thereby remove a potential low-intensity mechanical input that could modulate transport dynamics. We stress, however, that this is a secondary and testable proposition: IMV-related effects, if present, should be evaluated as additive modulation after accounting for sleep architecture, arousals, and hypoxia-related confounders. This also highlights a key confounding structure: sleep quantity/architecture and sleep-disordered breathing (e.g., arousals, intermittent hypoxia) can influence both clearance physiology and the magnitude/pattern of nocturnal cranio-cervical vibration exposure. Therefore, any evaluation of IMV contributions must explicitly control for polysomnographic sleep metrics and OSA-related indices, and should test whether quantified IMV dose explains additional biomarker variance beyond sleep architecture itself. This discrimination logic is reflected in our proposed study designs using concurrent sleep assessment and cranial/neck accelerometry.

### 3.2 Rhythmic stimulation as precedent for mechanically sensitive clearance pathways

#### 3.2.1 Rationale and interpretive boundary

Because direct experimental evidence for speech-like IMVs is currently lacking, we present externally applied rhythmic modalities only as precedent that clearance-related physiology can be modulated by appropriately constrained oscillatory inputs. Importantly, these modalities remain fully compatible with alternative mechanisms (e.g., neurovascular coupling, arousal/sleep architecture, immune modulation, or BBB permeability), and therefore should not be interpreted as direct evidence that everyday self-vocalization or benign upper-airway vibration produces the same effects. Instead, they motivate discriminating tests (Sections 5 and 6.3) in which mechanical dose is quantified and controlled.

##### 3.2.1.1 40-Hz sensory/gamma stimulation: supportive findings and ongoing debate

Multiple studies suggest that inducing gamma-range activity can reduce Aβ-related pathology in mouse models and can be feasible in humans. Early GENUS work reported reduced amyloid burden and microglial changes in AD mouse models, and subsequent studies extended GENUS to multi-sensory paradigms with glymphatic/meningeal lymphatic signatures that were mechanistically linked to amyloid removal in mice (Iaccarino et al., 2016; Martorell et al., 2019; Chan et al., 2022; Murdock et al., 2024). Human studies remain largely feasibility-oriented, and the clinical significance is still being established (Chan et al., 2025).

However, the field is not uniformly positive: rigorous recordings in AD-model mice have reported that 40-Hz visual flicker may fail to entrain native gamma oscillations in deeper structures and may not suppress Aβ under certain conditions (Soula et al., 2023). We therefore treat gamma stimulation as an instructive precedent that rhythmic drive can engage clearance-relevant pathways, while recognizing that effectiveness depends on stimulation modality, circuitry engaged, and disease context, and that the pathway need not be “mechanical” per se.

##### 3.2.1.2 Ultrasound and focused ultrasound: clearance signals with distinct mechanisms

Low-intensity ultrasound paradigms have reported amyloid reduction and behavioral benefits in AD-model mice in multiple studies, often discussed in relation to microglial responses, vascular effects, or altered transport dynamics (Leinenga and Götz, 2015). In humans, low-intensity focused ultrasound has been repeatedly shown to open the BBB transiently and reversibly, enabling enhanced delivery of anti-amyloid therapies (Lipsman et al., 2018). In a recent study combining repeated BBB opening with Aducanumab, amyloid reduction appeared greater in targeted regions than in contralateral control regions, while emphasizing the preliminary nature and limited sample size (Rezai et al., 2024).

These ultrasound modalities differ profoundly from everyday IMVs in frequency (kHz–MHz vs. tens–hundreds of Hz), focality, and likely dominant mechanisms (BBB permeability and immune effects may dominate). We cite them only to illustrate that clearance and plaque biology can be responsive to constrained physical/mechano-acoustic inputs, not to claim mechanistic equivalence with IMVs.

##### 3.2.1.3 Whole-body vibration: emerging but non-specific cognitive signals

Whole-body vibration (WBV) has shown emerging cognitive or executive-function signals in some small trials and reviews(Wen et al., 2023; Seok et al., 2025), particularly in older adults or populations with limited mobility, but protocols and effect sizes vary and mechanisms remain uncertain (Seok et al., 2025; Shantakumari and Ahmed, 2023; Prates et al., 2025). The relevance to IMVs is therefore indirect: WBV does suggest that low-intensity oscillatory mechanical exposure can be tolerated and may modulate brain-relevant outcomes, but current evidence does not isolate clearance-specific pathways and is susceptible to confounding (exercise, arousal, sleep quality).

##### 3.2.1.4 Cervical lymphatic mechanical manipulation: a direct link to CSF outflow

Particularly relevant to a “mechanical sensitivity” argument, recent work reported that gentle, force-regulated non-invasive manipulation of superficial cervical lymphatics through intact skin can markedly increase CSF outflow and rescue age-related drainage impairment in mice (Jin et al., 2025). This provides a concrete example that low-intensity mechanical input at peripheral lymphatic interfaces can causally modulate CSF drainage, a pathway directly adjacent to glymphatic/meningeal lymphatic clearance concepts.

##### 3.2.1.5 Interpretive synthesis

Table 1 summarizes the externally applied rhythmic modalities discussed above, highlighting for each modality the typical exposure characteristics, the dominant plausible (non-IMV) mechanisms, and—critically—why these findings should be treated as precedent rather than direct evidence for speech-like IMVs. Across these modalities, the shared and conservative inference is that clearance-adjacent physiology (glymphatic/CSF transport, meningeal/lymphatic drainage, immune/glial states, and vascular dynamics) can be modulated by appropriately constrained rhythmic inputs. This is compatible with—but does not prove—the possibility that naturally occurring IMVs from self-vocalization or benign upper-airway vibration could serve as a low-intensity physiological analogue. For this reason, we treat these literatures as precedent and mechanistic plausibility, while placing the evidentiary burden on direct, dose-quantified, confounder-controlled tests proposed later.

**Table 1:** Externally applied rhythmic modalities cited as precedent for mechanically sensitive clearance-adjacent physiology (indirect evidence for IMVs).

Modality	Typical frequency/“dose” characteristics	Delivery/exposure context	Reported effects relevant to clearance/pathology	Dominant plausible mechanisms (non-exclusive)	What this *supports* for the IMV hypothesis (conservative interpretation)	Why this is *not* direct evidence for speech-like IMVs (key limitations/confounds)	References
40-Hz sensory / gamma entrainment	~40 Hz rhythmic drive; dose depends on intensity, session duration, and multimodal coupling	Visual/auditory/tactile stimulation; central neural entrainment	Reduced Aβ burden and microglial remodeling in some AD mouse studies; feasibility/biomarker exploration in humans	Neural entrainment; microglial/immune modulation; neurovascular coupling; sleep/arousal changes; network-level effects	Shows that rhythmic drive can modulate clearance-adjacent physiology, and motivates testing whether mechanical vibration is one possible co-factor	Frequency and delivery differ from IMVs; effects can arise without mechanical transmission; mixed/negative findings exist depending on paradigm; not specific to mechanochemical mixing	Iaccarino et al. (2016), Martorell et al., 2019; Chan et al. (2022), Murdock et al. (2024), Chan et al., 2025, and Soula et al. (2023)
Whole-body vibration (WBV)	Typically low-frequency mechanical oscillation; dose varies by platform and posture	Peripheral mechanical exposure; systemic effects	Emerging cognitive signals in some small trials/reviews	Arousal, circulation, vestibular/autonomic effects; exercise-like systemic changes; sleep effects	Indicates that low-intensity oscillatory mechanical exposure can be tolerated and may influence brain-relevant outcomes	Not clearance-specific; high confounding risk (activity, arousal, placebo); heterogeneity in protocols/outcomes; mechanism uncertain	Wen et al. (2023), Seok et al. (2025), Shantakumari and Ahmed (2023), and Prates et al. (2025)
Low-intensity ultrasound / focused ultrasound (FUS)	kHz–MHz; focal high-frequency mechano-acoustic input	Localized brain targeting; sometimes BBB opening	Aβ reduction / behavioral signals in mice; BBB opening feasibility in humans; potential synergy with antibodies	BBB permeability; immune/glial changes; vascular effects; acoustic radiation force; local fluid dynamics	Shows that physical/mechano-acoustic inputs can influence plaque biology and transport-related pathways	Frequency regime is fundamentally different; dominant mechanism may be BBB/immune; not analogous to natural IMVs; intervention is device-driven and focal	Leinenga and Götz (2015), Lipsman et al. (2018), and Rezai et al. (2024)
Gentle cervical lymphatic mechanical manipulation	Low-force, rhythmic peripheral mechanical input; dose set by force and duration	Superficial cervical lymphatic interface; intact skin	Increased CSF outflow; improved drainage in aged mice	Lymphatic pumping mechanics; altered CSF/lymphatic interface dynamics	Provides a more direct “mechanical input → CSF drainage” precedent adjacent to glymphatic/meningeal lymphatics	Still not cranial IMVs; mouse data; translation uncertain; requires careful separation from respiration/sleep/arousal confounds	Jin et al. (2025)

This table summarizes externally applied rhythmic modalities as precedent that clearance-adjacent physiology may be sensitive to constrained oscillatory inputs. These studies are not presented as direct evidence that speech-like intrinsic mechanical vibrations (IMVs) enhance Aβ clearance. Many findings have alternative explanations (neurovascular coupling, arousal/sleep architecture, BBB effects, immune modulation), and therefore the hypothesis must be evaluated primarily via discriminating tests with quantified mechanical dose and matched non-mechanical controls (Sections 5 and 6.3).

### 3.3 Music, active singing, and dementia outcomes

Music-based interventions improve mood, behavior, and sometimes cognition in dementia (Leggieri et al., 2019; Ting et al., 2024). Trials in which participants actively sing often report larger benefits than those emphasizing passive listening or usual care, including gains in memory and quality of life for both patients and caregivers (Särkämö et al., 2013; Särkämö et al., 2016; Bleibel et al., 2023).

These effects are typically interpreted through cognitive, emotional, and social mechanisms. We do not dispute those pathways. We suggest an additional biophysical nuance: active vocalization imposes strong, proximal cranio-facial vibrations that are largely absent during passive listening, potentially providing an IMV “top-up” aligned with clearance-oriented improvements.

Crucially, this mechanical interpretation is testable: future trials can include (i) active vocalization versus (ii) matched non-vocal cognitive engagement and (iii) dose-matched external vibro-stimulation calibrated by skull accelerometry, enabling explicit separation of vibrational dose from social, emotional, and cognitive drivers.

### 3.4 Hearing impairment and dementia risk

Age-related hearing loss is a robust, dose-dependent risk factor for dementia, with higher levels of loss associated with substantially elevated hazard ratios (Lin et al., 2011; Livingston et al., 2020). Randomized evidence indicates that treating hearing loss can slow cognitive decline in higher-risk older adults (Lin et al., 2023; Liu, 2024).

Mainstream interpretations emphasize increased cognitive load, social isolation, and sensory deprivation. Our hypothesis introduces a testable mechanical co-pathway: individuals with substantial hearing impairment may speak less and vocalize less over time, reducing their accumulated IMV dose and slightly biasing clearance against Aβ. This idea does *not* conflate Deaf identity with disease risk and does not claim that mechanical factors are primary; it simply adds a modifiable behavioral dimension that may coexist with psychosocial mechanisms.

### 3.5 Snoring as a common source of nocturnal cranio-cervical vibration

Upper-airway vibration during sleep (often labeled “snoring” or stertor) has been reported across multiple mammalian species (Lonergan et al., 1998; Toth and Bhargava, 2013; Beck et al., 1995; Chopra et al., 2016). Here we treat it primarily as an exposure source of cranio-cervical vibration, not as evidence of an adaptive trait. We therefore do not argue that snoring is evolutionarily conserved because it improves clearance. Instead, we make a narrower and testable point: when upper-airway vibration occurs without hypoxia or marked sleep fragmentation, it may contribute to the nocturnal IMV exposure spectrum. This exposure can be quantified (e.g., via neck/skull accelerometry and respiratory indices) and evaluated while carefully controlling for obstructive sleep apnea, intermittent hypoxia, arousals, and other confounders that are independently linked to amyloid and cognitive outcomes.

### 3.6 Modeling support

As a first check on physical plausibility, we modeled sound propagation and intracranial acoustic fields using finite element (FEM) simulations in COMSOL Multiphysics (see Supplementary Figures S1, S2). We compared three conditions with calibrated sound pressure levels (65 dB SPL), using different reference points: (i) passive listening to a sound source 1 m away, adjusted to yield 65 dB SPL at the ear; (ii) self-initiated vocalization (“singing”), adjusted to yield 65 dB SPL at 1 m from the mouth; and (iii) closed-mouth humming (same calibration as singing). In the 20–400 Hz range, the average acoustic pressure in the brain during self-vocalization was on the order of 60–70-fold higher (≈36 dB) than during passive listening, indicating much stronger near-field vibrational drive for the same perceived loudness. The ratio peaked around 760 Hz, where self-vocalization produced ~550-fold higher average intracranial pressure (≈55 dB) than passive listening, consistent with resonant properties of the vocal tract, although such peaks are likely damped *in vivo* by tissue absorption and other losses. At higher frequencies the relative advantage of self-vocalization declined as standing-wave patterns changed. These simplified simulations do not capture the full complexity of skull and tissue mechanics, but they support the basic claim that self-generated vocal sounds couple substantially more mechanical energy into the brain than equally loud external sounds heard at a distance.

We emphasize that the FEM results are intended as an order-of-magnitude plausibility and relative-coupling comparison, not a definitive prediction of in vivo intracranial pressure fields. The current model does not fully capture tissue heterogeneity, fluid–structure coupling at CSF interfaces, frequency-dependent damping in living tissues, or the potential influence of vascular pulsatility, all of which may alter attenuation and spatial distribution. Accordingly, a key next step is empirical validation using (i) standardized skull-surface accelerometry during controlled self-vocalization versus passive listening, and (ii) cranial phantoms with tissue-mimicking materials and a fluid layer approximating CSF to calibrate damping parameters and guide subsequent model refinement.

## 4 Evidence that appears inconsistent and how the hypothesis can be tested

### 4.1 Voice-heavy occupations

A simple prediction might be that vocally intensive professions (teachers, singers, clergy, broadcasters) would exhibit lower AD incidence or later onset. To date, however, there are no large, well-controlled cohort studies that specifically quantify “vocal load” and relate it to dementia risk. Occupational analyses typically emphasize education, cognitive complexity, and socioeconomic factors (Hyun et al., 2022), and “musician” categories often include both singers and instrumentalists (Balbag et al., 2014; Arafa et al., 2022). Moreover, many voice-heavy jobs involve chronic noise exposure and associated hearing loss—risk factors in their own right (Cantuaria et al., 2024; Cloeren et al., 2025; Zhang et al., 2025).

We therefore view the absence of a clear occupational signal as a reflection of measurement and confounding limitations, not strong disconfirmation. The correct test is not crude job category, but direct, wearable measurement of vocal and vibration dose, with careful adjustment for hearing loss, sleep/OSA, education, and vascular/metabolic risk.

### 4.2 Obstructive sleep apnea, loud snoring, and amyloid burden

AD cohorts have higher rates of obstructive sleep apnea (OSA), and OSA is associated with increased amyloid and tau burden, adverse CSF profiles, and elevated dementia risk (Emamian et al., 2016; Maimon and Hanly, 2010; Bubu et al., 2020). Snoring intensity correlates with OSA severity. At first glance, this seems to contradict the idea that upper-airway vibrations might be beneficial.

The IMV framework draws a distinction between:

- Physiological snoring: low-intensity vibration during stable, non-fragmented sleep with preserved oxygenation and modest pressure swings.
- Pathological OSA-associated snoring: vibration embedded in a pattern of repeated airway collapse, intermittent hypoxia, large intrathoracic pressure changes, arousals, and cardiovascular stress.

We anticipate that in OSA, any potential benefit from vibration is outweighed by the harms of hypoxia and fragmentation. Consistent with this, treating OSA with CPAP improves slow-wave activity, sleep consolidation, cognition, and several biomarker measures, even though mechanical vibration may be reduced. A central, testable prediction is that after controlling for oxygen desaturation and arousal indices, vibration dose from non-hypoxic snoring (or from safe daytime vocalization or vibro-stimulation) will correlate with better clearance proxies, whereas vibration delivered in the context of OSA will not.

## 5 Hypothesis testing and experimental strategy

To empirically evaluate the IMV–clearance hypothesis, we propose a coordinated program spanning four complementary domains: interventional clinical trials, tightly controlled human laboratory paradigms, mechanistic preclinical studies, and prospective epidemiological cohorts. Across these approaches, the core strategy is to (i) quantify IMV dose using voice/vibration dosimetry and controlled vibro-stimulation or structured self-vocalization, (ii) manipulate IMV exposure acutely and chronically, and (iii) track convergent clearance-relevant outcomes: perivascular/glymphatic imaging proxies, BBB/meningeal-lymphatic markers, CSF and plasma Aβ dynamics, sleep/OSA architecture, and cognitive performance.

### 5.1 Testable and discriminating predictions

A practical near-term test would be a within-subject crossover study in middle-aged adults:

- Condition A: quiet rest following partial sleep restriction.
- Condition B: similar rest, but with brief periods of closed-mouth humming or safe cranio-cervical vibro-stimulation.

Primary outcomes could include MRI-based proxies of perivascular and glymphatic flow and short-term plasma or CSF Aβ dynamics. Sleep architecture and cardiovascular parameters would be monitored and controlled. The specific prediction is that Condition B will show more favorable changes in clearance-linked metrics than Condition A, in the absence of differences in cognitive or social engagement.

### 5.2 Clinical trials

In individuals with subjective cognitive decline, mild cognitive impairment, or early AD, randomized trials could compare:

- Daily self-vocalization regimens (e.g., structured humming or singing practice), or
- Oral or bone-conduction vibro-stimulation tuned to speech-like low frequencies, against sham or active controls such as passive listening with matched social contact. Outcomes would include cognitive and functional measures, sleep metrics, and imaging or fluid biomarkers of Aβ and clearance. Because an optimal IMV window is not yet established, initial protocols should be anchored to physiological, naturalistic vibration ranges (measured during everyday vocalization) with conservative titration and careful monitoring of sleep quality, arousals, and respiration/hypoxia indices.

An important translational question is how IMV modulation interacts with Aβ-targeting therapies. For example, does increasing IMV exposure before or during treatment with monoclonal antibodies enhance plaque removal, reduce ARIA risk at a given dose, or allow effective treatment at lower doses?

### 5.3 Preclinical studies

In AD model mice, mechanistic experiments could address:

- Necessity: whether modest vocalization deprivation or dampening of head/neck vibrations (while minimizing stress and controlling respiration) worsens Aβ burden or glymphatic function.
- Sufficiency: whether daily head/neck vibro-stimulation (broadband or at defined frequencies such as 40 Hz) reduces plaque load, improves glymphatic flux and AQP4 polarization, increases meningeal-lymphatic outflow, and benefits cognition.
- Mediation: whether disrupting perivascular pumping or AQP4 function abolishes the effects of IMV-like stimulation.
- Feasibility: before large *in vivo* studies, speech-like vibration waveforms (matched to measured IMV spectra) can be applied to tractable systems—such as tracer transport in gels/porous media, ex vivo brain-slice preparations, or CSF-mimicking microfluidic/phantom models—to directly test whether calibrated low-frequency vibration measurably increases dispersion/mixing, perivascular-like transport surrogates, or effective tracer flux compared with matched no-vibration controls.

### 5.4 Prospective cohorts

Prospective cohorts equipped with wearable voice and vibration sensors, detailed sleep and OSA assessments, audiometry, and longitudinal cognitive testing could directly examine whether natural variation in IMV dose predicts amyloid PET signal and dementia risk. In addition to voice-activity metrics, cohorts could incorporate direct cranial vibration dosimetry (e.g., tri-axial accelerometry at standardized skull sites such as mastoid/temporal region and forehead/vertex, alongside a neck-surface sensor) to quantify transmitted IMV dose in frequency bands and cumulative metrics. Rigorous adjustment for confounding factors (sleep quality, OSA, hearing status, education, and vascular/metabolic risk) would be essential. Such data would address limitations of previous occupational and lifestyle proxies.

## 6 Discussion

### 6.1 Summary and interpretive context

This hypothesis paper proposes that age-related reductions in low-intensity mechanical vibrations of the head and neck—arising primarily from day-to-day vocalization and benign upper-airway motion—may contribute to Aβ clearance failure in mid-life. We suggest that these intrinsic mechanical vibrations can, in principle, enhance interstitial mixing, perivascular and glymphatic transport, and encounter rates at BBB and meningeal-lymphatic interfaces. In a setting where Aβ production is relatively stable, a gradual loss of this mechanical drive could be one of several factors that make it easier for Aβ to accumulate and form plaques.

The proposal is deliberately modest. It does not claim that IMVs are a primary cause of AD, nor that mechanical effects replace genetics, inflammation, vascular injury, or tau pathology. Instead, IMVs are treated as a potential modifier of clearance efficiency that operates alongside these established mechanisms. The value of the framework is that it connects everyday behaviors (how much people speak or sing, how long and how well they sleep) to biophysical processes (fluid flow and solute transport) that are already known to matter in AD, and it generates a set of concrete experiments that can be performed with current methods.

### 6.2 Relationship to existing AD frameworks and amyloid-β therapies

Within amyloid-centered frameworks, the central question is not whether Aβ is important, but why and when normal homeostatic control is lost. Recent emphasis on clearance has drawn attention to glymphatic and meningeal-lymphatic flow, arterial pulsatility, sleep architecture, and receptor-mediated transport at the BBB. IMVs fit naturally into this clearance-centric view as one more, often overlooked, source of low-frequency mechanical input.

For current therapies that target aggregated Aβ with monoclonal antibodies, the hypothesis has two potential implications. First, chronic differences in IMV exposure—shaped by sleep habits, vocal behavior, and cranio-cervical biomechanics—might contribute to variability in treatment response, for example by influencing how readily the brain can handle mobilized Aβ. Second, if IMV augmentation proves feasible and safe, it could act as a non-pharmacologic adjunct that modestly increases clearance capacity, particularly in preclinical or prodromal stages.

At the same time, it is important to recognize how early the field is in this area. The evidence assembled here is indirect: sleep physiology, 40-Hz stimulation, ultrasound, music and singing interventions, hearing-loss epidemiology, and evolutionary arguments can all be interpreted through other, more established mechanisms. No trial has yet tested whether IMV-focused interventions alter Aβ biomarkers or clinical trajectories. Any discussion of clinical use must therefore remain conditional until such studies are done.

### 6.3 Testable predictions and falsifiability

The IMV–clearance hypothesis leads to several specific, testable predictions:

- In short-term human experiments, adding brief periods of self-vocalization or safe cranio-cervical vibro-stimulation after sleep loss should produce measurable changes in clearance-linked metrics—such as perivascular/glymphatic imaging proxies or short-term plasma/CSF Aβ dynamics—compared with matched quiet-rest controls.
- In controlled benchtop or ex vivo systems (e.g., gels, brain slices, or CSF-mimicking phantoms), applying calibrated speech-like vibration waveforms at matched doses should measurably increase tracer dispersion or effective transport rates compared with no-vibration controls; failure to observe such effects would argue against the proposed mixing/transport mechanisms.
- In AD model animals, dampening head-and-neck vibrations should worsen Aβ burden or glymphatic function, whereas restoring or augmenting gentle vibrations should improve these outcomes in a manner that depends on intact perivascular and AQP4-dependent pathways.
- In prospective cohorts with wearable dosimetry, higher long-term IMV dose (after adjusting for sleep/OSA, hearing loss, education, and vascular/metabolic risk) should predict more favorable amyloid PET and cognitive trajectories.
- In patients receiving monoclonal antibodies, pre-treatment IMV exposure or experimentally increased IMV dose should correlate with more efficient plaque removal or better clinical response at a given level of drug exposure, if IMVs meaningfully modulate clearance.

If these patterns are not observed in well-designed studies, the hypothesis should be revised or discarded. The framework is intended to be straightforward to test and, if necessary, to refute.

### 6.4 Limitations of the hypothesis

Several limitations deserve emphasis. First, almost all of the supporting evidence is indirect. The literatures on sleep and clearance, 40-Hz stimulation, ultrasound, music interventions, hearing loss, and snoring each have their own, well-grounded mechanistic explanations. The IMV perspective is an additional layer, not a replacement, and it should be weighed accordingly.

Second, there is a risk of over-interpreting observational associations. The link between hearing loss and dementia, for example, is already well explained by cognitive, social, and sensory factors; any mechanical pathway via reduced self-vocalization is speculative at present. Similarly, the prevalence of snoring-like sounds in mammals does not by itself establish a beneficial role; many evolutionary scenarios are possible.

Third, some aspects of the topic are socially and clinically sensitive. Discussions involving Deaf communities, hearing impairment, and snoring must avoid implying causality or stigma that is not supported by data. The present framework does not claim that Deaf identity or cultural practices cause dementia, nor does it recommend snoring as a health strategy. It distinguishes between modest-intensity vibrations, which might be delivered via vocalization or devices, and pathological patterns such as OSA, where hypoxia and sleep fragmentation are clearly harmful. Future empirical work should be designed with input from affected communities and with careful ethical framing.

Finally, any effects of IMVs are likely to be modest at the individual level. IMVs are proposed as one factor among many—genetic, vascular, metabolic, inflammatory, and lifestyle-related—that shape overall risk. Even if the hypothesis is supported, meaningful public-health impact would probably require combining IMV-related strategies with other established interventions rather than relying on them alone.

### 6.5 Translational and therapeutic implications

Despite these caveats, the hypothesis points to several translational directions that can be explored in a controlled manner. Behavioral programs that emphasize structured self-vocalization—such as humming or singing practice—could be evaluated in randomized trials for their effects on clearance-linked biomarkers, cognition, and quality of life in at-risk or early-stage populations. These interventions would need carefully designed control conditions to separate mechanical contributions from cognitive and social ones.

In parallel, device-based approaches using oral or bone-conduction vibro-stimulation tuned to speech-like frequencies could offer a controllable way to deliver IMV-like input, particularly for people with vocal limitations. Early-phase trials should focus on safety, tolerability, and mechanistic endpoints (perivascular/glymphatic imaging, sleep architecture, autonomic responses) before any claims about clinical efficacy are made. At present, there is no basis to recommend unsupervised vibration regimens as preventive or therapeutic measures.

With respect to Aβ-targeting therapies, validated IMV-based strategies might eventually play three roles: (i) primary prevention by slightly prolonging the period during which endogenous clearance keeps pace with production; (ii) adjunctive support to increase the efficiency of pharmacologic plaque removal or to reduce required drug doses; and (iii) rehabilitative support in later stages, integrated with music-based and cognitive interventions that already have some evidence in dementia care. These possibilities are speculative, but they provide a concrete agenda for translational research.

### 6.6 Future directions

The next step is to move from conceptual synthesis to direct tests. Short-term human experiments that manipulate IMV exposure under controlled conditions, mechanistic animal studies that examine necessity and sufficiency, and cohort designs that directly measure vocal and vibration dose are all feasible with current methods. More detailed computational models of solute transport that incorporate realistic cranio-cervical mechanics could refine predictions and identify the ranges of vibration amplitude, frequency, and duration where meaningful effects are plausible.

More broadly, the IMV framework encourages a more systematic treatment of mechanical forces in models of brain homeostasis and neurodegeneration. Even if IMVs themselves prove to have only a minor role, clarifying their impact will help refine our understanding of the production–clearance balance at the heart of the amyloid debate and may influence how emerging therapies are combined and timed.

## 7 Conclusion

We propose that age-related declines in vibration exposure constitute a missing upstream factor in Aβ clearance failure. By placing an explicitly defined IMV dose within a mechanochemical cascade—from mid-life changes in behavior and sleep to reduced transport, Aβ retention, tau facilitation, and neurodegeneration—this framework offers a parsimonious, testable account of why pathology accelerates when it does. The hypothesis generates clear predictions, practical laboratory and clinical tests, and low-burden interventions (self-vocalization, safe vibro-stimulation) that could augment existing prevention and pharmacologic strategies. Validating—or falsifying—this proposal would meaningfully advance our understanding of AD onset and open accessible avenues to bolster brain proteostasis across the lifespan.

## Data Availability

The original contributions presented in the study are included in the article/Supplementary material, further inquiries can be directed to the corresponding author.
