# Supplementary material for: Intrinsic mechanical vibrations as a missing dimension in amyloid-β clearance: a mechanochemical hypothesis for Alzheimer’s disease
Source: Front Aging Neurosci. 2026 Jan 20;18:1749562. doi: 10.3389/fnagi.2026.1749562 (PMC12864501; doi:10.3389/fnagi.2026.1749562)
Supplement: Supplementary file 1 [file Data_Sheet_1.pdf]

Supplementary Information

Intrinsic mechanical vibrations as a missing dimension in amyloid-β clearance: a mechanochemical hypothesis for Alzheimer's disease

Author: Xiaochen Lai[1*]
[1] School of Future Technology, Nanjing University of Information Science & Technology, Nanjing, China

Correspondence: xchlai@nuist.edu.cn

FEM Study on Acoustic Brain Vibrations Induced by Vocalization

To provide quantitative support for our argument that self-initiated vocalization produces stronger brain vibrations compared to passive listening, we conducted a series of numerical simulations using finite element method (FEM) acoustic field modeling with COMSOL Multiphysics. Detailed configurations and further analysis of the simulations are provided at the end of this part. The primary aim of the FEM study was to compare the vibrational impact on the brain between passive listening, singing, and humming. We report the modeled intracranial acoustic-pressure magnitude (|p|) and its spatial average within the brain domain under three sound-generation scenarios.

We simulated the acoustic pressure distribution in the brain for three conditions:

1. Passive listening with a sound source 1 meter away, adjusted to achieve a fixed sound pressure level (SPL) of 65 dB (reference 20 μPa) at the ear.
2. Self-initiated vocalization (singing) with source adjusted to produce a SPL of 65 dB at a distance of 1 meter.
3. Humming, same configuration to singing but with the mouth closed to simulate humming.

The simulations revealed significant differences in the acoustic pressure distributions and magnitudes among the three conditions. In the case of passive listening, the acoustic pressure in the brain is relatively low (Figure S1c and S1d). In contrast, self-vocalization (Figure S1a and S1b) generates much higher acoustic pressures throughout the brain. The peak acoustic pressures during self-vocalization can be up to hundreds of times greater than those during passive listening, indicating a much stronger vibrational impact.

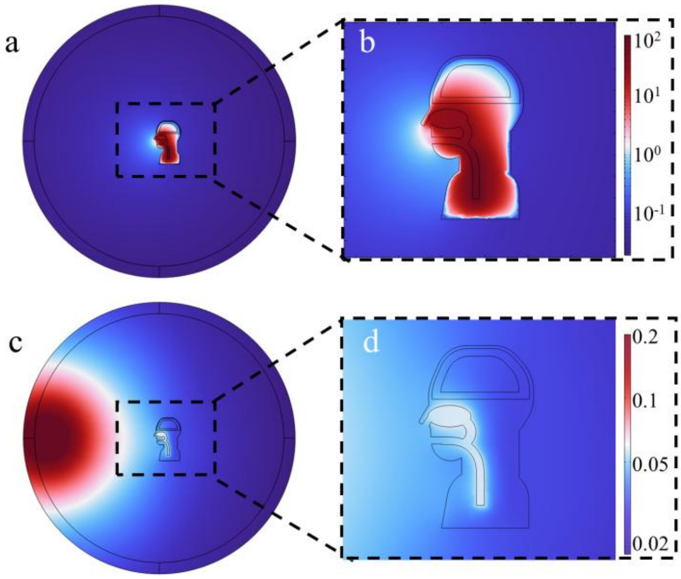

Figure S1: (a) Acoustic pressure distribution in the brain during self-vocalization (singing) at 200 Hz. (b) Zoomed-in view of acoustic pressure distribution during self-vocalization. (c) Acoustic pressure distribution in the brain during passive listening at 200 Hz. (d) Zoomed-in view of acoustic pressure distribution during passive listening.

The FEM simulation results provide a detailed analysis of the average acoustic pressure in the brain for self-vocalization compared to passive listening across a range of frequencies. In the frequency range of 20-400 Hz, the enhancement effect of singing is relatively uniform, with the average acoustic pressure being approximately 67.6 times (36.54 dB) higher than that of passive listening. This consistent enhancement indicates that self-vocalization significantly boosts brain vibrations in this lower frequency range.

This observation is further quantified in Figure S2a, which plots the average acoustic pressure in the brain across different frequencies. The blue line representing self-vocalization consistently shows higher values compared to the green line for passive listening. Notably, there are peaks in the passive listening chart around 500 Hz, which could be attributed to the resonant frequencies of the external auditory system, where sound waves are naturally amplified.

In addition to the 500 Hz resonance, Figure S2b reveals a significant peak at 760 Hz, where the ratio of self-vocalization to passive listening reaches its maximum, with a staggering 556.9 times (or 54.9 dB) increase. This peak is likely due to the complex resonant characteristics of the vocal tract, which includes the mouth and nasal cavities forming a double open-ended resonator. However, it is important to consider that in practical scenarios, the actual increase might not be as pronounced due to the absorption of sound waves by soft tissues and the conversion of acoustic energy into heat.

At higher frequencies, the enhancement effect of self-vocalization on brain vibrations diminishes significantly. This reduction can be attributed to the changes in standing wave patterns. Consequently, the effectiveness of singing in inducing brain vibrations decreases as the frequency increases beyond the resonance peaks.

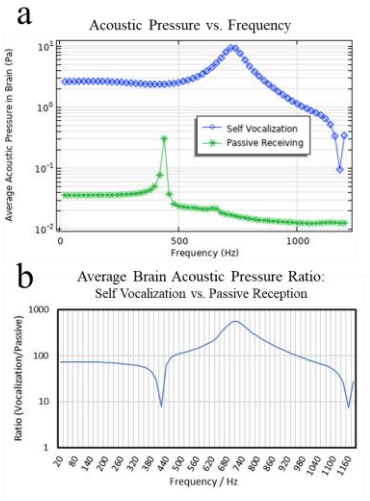

Figure S2: (a) Average acoustic pressure in the brain as a function of frequency for self-vocalization and passive listening. (b) Ratio of average brain acoustic pressure for self-vocalization versus passive listening across frequencies.

Comparing singing (mouth open) and humming (mouth closed) in Figure S3a and S3b, the simulations show that humming leads to deeper penetration and even higher acoustic pressures in the brain. The closed-mouth condition confines the sound energy, enhancing vibrational transmission to the brain. The frequency for humming was tested up to 500 Hz since it is challenging to generate high-

frequency sounds with the mouth closed. Moreover, we are particularly interested in low frequencies, which cover the frequency range that is relevant to snoring.

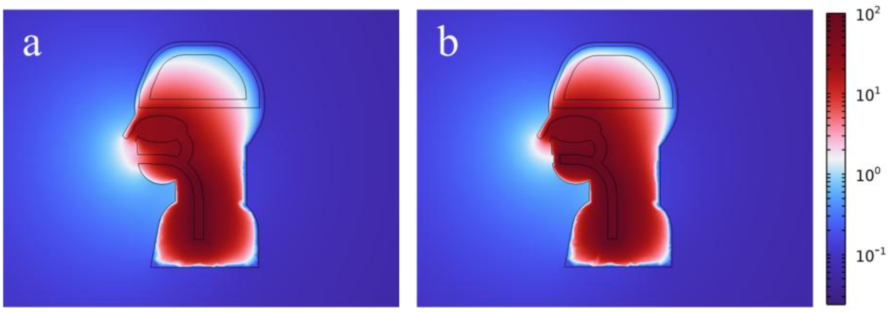

Figure S3: (a) Acoustic pressure distribution in the brain during singing (mouth open) at 200 Hz. (b) Acoustic pressure distribution in the brain during humming (mouth closed) at 200 Hz.

Figure S4a illustrates the average acoustic pressure for singing and humming across different frequencies. Humming consistently shows higher pressures, particularly at lower frequencies. This may be due to the increased resonance and energy concentration within the oral and nasal cavities when the mouth is closed. The ratio graph in Figure S4b supports this observation, indicating that humming can be up to 1.8 times more effective than singing.

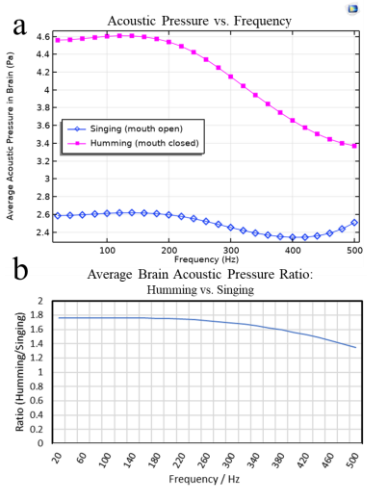

Figure S4: (a) Average acoustic pressure in the brain as a function of frequency for singing and humming. (b) Ratio of average brain acoustic pressure for humming versus singing across frequencies.

Analyzing the data, we observe that acoustic pressure and the ratio of vocalization to passive listening change with increasing frequency. For self-vocalization, acoustic pressure generally decreases with higher frequencies, except for specific resonant peaks. This trend suggests that lower frequencies might be more effective in generating substantial brain vibrations. The relatively stable and higher modeled pressures during humming indicate stronger intracranial coupling under the closed-mouth configuration. This finding is relevant for prioritizing which vibration patterns to measure and replicate experimentally, rather than implying therapeutic efficacy.

Taken together, these FEM simulations suggest that self-initiated vocalization—especially humming—can plausibly produce higher intracranial acoustic pressures than passive listening under the modeled assumptions, motivating empirical validation with direct vibration measurements in phantoms and in vivo settings before any clearance-related claims are made.

Materials & Methods

FEM Model Description

The finite element method (FEM) simulations were conducted using COMSOL Multiphysics. The half-body human model geometry was imported, with detailed structures for the nasal cavity, oral cavity, nasopharynx, and larynx drawn accordingly. To simplify the design and reduce computational load, the oral cavity, nasopharynx, and larynx were represented using cylindrical hollow structures with a diameter of 1.6 cm. The nasal cavity was approximated as an ellipsoid with a volume of 80 mL. The mouth opening area was approximately 4 cm^2, and the nasal openings were about 1.7 cm^2. The skull and brain were modeled to match the head's geometry but at smaller scales, with the skull thickness set to about 1 cm (Figure S5a).

Simulation Module

The Pressure Acoustics, Frequency domain was employed to study the interaction of sound within human tissues. This module was chosen for its simplicity and reliability, as well as its computational speed. However, it is a linear model and cannot fully account for the complexities of acoustic-solid coupling, where sound-induced deformations in solid boundaries alter the acoustic field. While multi-physics coupled models can address this issue, their computational demands are exceedingly high, making them impractical for our study's scope.

Material Properties

The materials used in the simulations included air, human tissue, skull bone, and brain tissue. The acoustic properties of these materials were defined as follows:

- Air: ρ = 1.21 kg/m^3, c = 343 m/s
- Human Tissue: ρ = 1050 kg/m^3, c = 1540 m/s
- Skull Bone: ρ = 1900 kg/m^3, c = 4080 m/s
- Brain Tissue: ρ = 1050 kg/m^3, c = 1540 m/s

Source Configuration and Parameter Scanning

Two monopole sources were respectively placed in the pharynx and 1 meter in front of the model to simulate both passive listening and active vocalization scenarios. Parameter scanning methods were utilized to control the activation and deactivation of the monopole sources, ensuring accurate simulation of the two different conditions.

Integration and Sampling Operators

Integration operator was positioned within the brain domain to calculate the spatial average of |p| within the brain domain. Additionally, 2 average operators were placed at the external monopole source point and 2 cm from human ear. These operators sampled reference point sound pressures to adjust system gain, ensuring consistent reference point sound pressures across comparative experiments.

External Boundary Conditions and Meshing

Perfectly Matched Layer (PML) boundary conditions were used along with swept hexagonal meshes to simulate an infinite space, preventing reflections from the boundaries that could interfere with the acoustic field (Figure S5b). This setup ensured that sound waves reaching the boundary would be absorbed without reflection, maintaining the integrity of the simulation results.

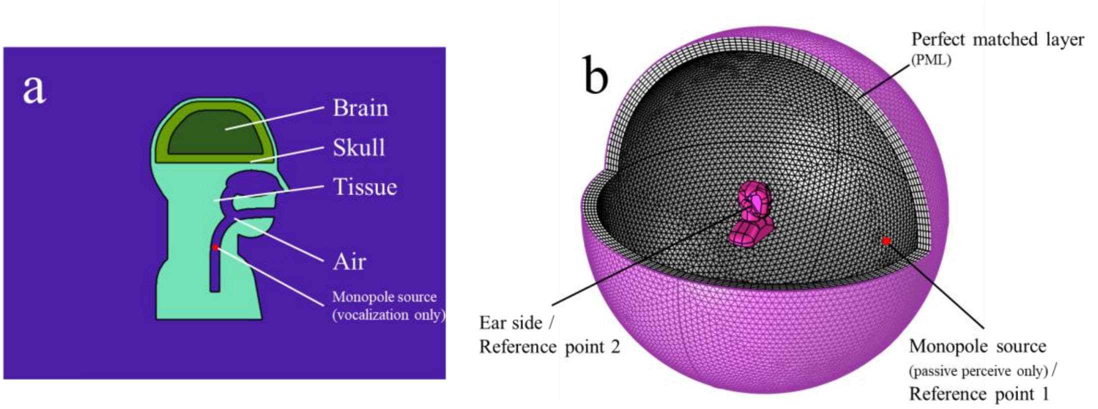

Figure S5. (a) Schematic of the half-body model detailing the brain, skull, tissue, and air cavities, as well as the monopole source locations. (b) FEM model setup showing the mesh configuration and placement of monopole sources for both vocalization and passive listening scenarios.

Simulation Results and Interpretation

The FEM Simulation Results for passive listening, self-vocalization (singing), and self-vocalization (humming) are shown in **Figure.S6-S8**.

Passive Listening

The FEM simulations for passive listening show acoustic pressure distribution in the brain across a range of frequencies from 20 Hz to 1200 Hz. It is noteworthy that the legend values for passive listening are significantly lower compared to self-vocalization conditions.

- **Low Frequencies (20–200 Hz):** At low frequencies, the acoustic pressure distribution is relatively uniform but low in magnitude. This indicates minimal penetration and resonance within the brain tissues, which is expected as lower frequencies generally do not resonate strongly in small, enclosed spaces like the human head.

- **Mid Frequencies (200–600 Hz):** In the mid-frequency range, especially around 400–500 Hz, there are noticeable increases in acoustic pressure. This range aligns with the resonant frequencies of the external auditory system, where sound waves are naturally amplified.

Standing waves and nodes can be observed, indicating areas of constructive and destructive interference.

- **High Frequencies (600–1200 Hz):** At higher frequencies, the acoustic pressure starts to diminish rapidly. This is due to the higher scattering of high-frequency waves by the tissues, leading to significant attenuation. The pressure distribution becomes more sporadic and less defined, highlighting the inefficacy of high frequencies in penetrating deeply into brain tissues.

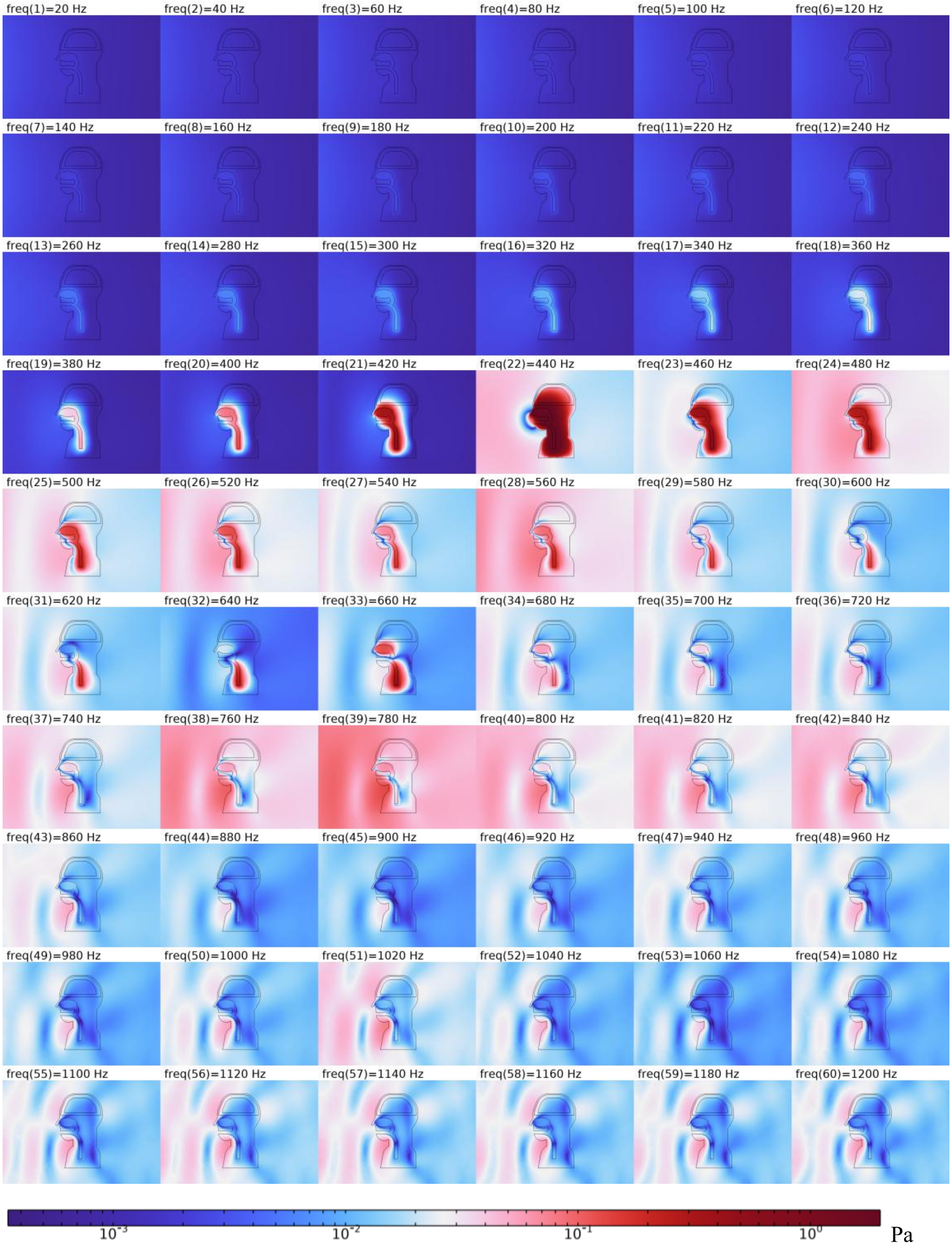

Figure S6: Acoustic pressure distribution in the brain during passive listening across frequencies from 20 Hz to 1200 Hz.

Self-Vocalization (Singing)

For self-vocalization (singing), the acoustic pressure distribution is shown across the same frequency range. The legend values are higher than in passive listening, indicating stronger vibrations.

- **Low Frequencies (20–200 Hz):** At low frequencies, the acoustic pressure is more concentrated and higher in magnitude compared to passive listening. This suggests better resonance and energy transmission within the brain, enhancing vibrational impact.
- **Mid Frequencies (200–600 Hz):** The mid-frequency range shows significant resonance, with peak pressures around 400–500 Hz, similar to passive listening but with much higher magnitudes. The formation of standing waves is more pronounced, indicating strong constructive interference and efficient energy transfer to the brain tissues.
- **High Frequencies (600–1200 Hz):** High-frequency behavior in self-vocalization shows a similar attenuation trend as passive listening, but the pressures remain higher. This indicates better penetration but still significant energy loss due to the change of stand wave pattern and scattering by the brain tissues.

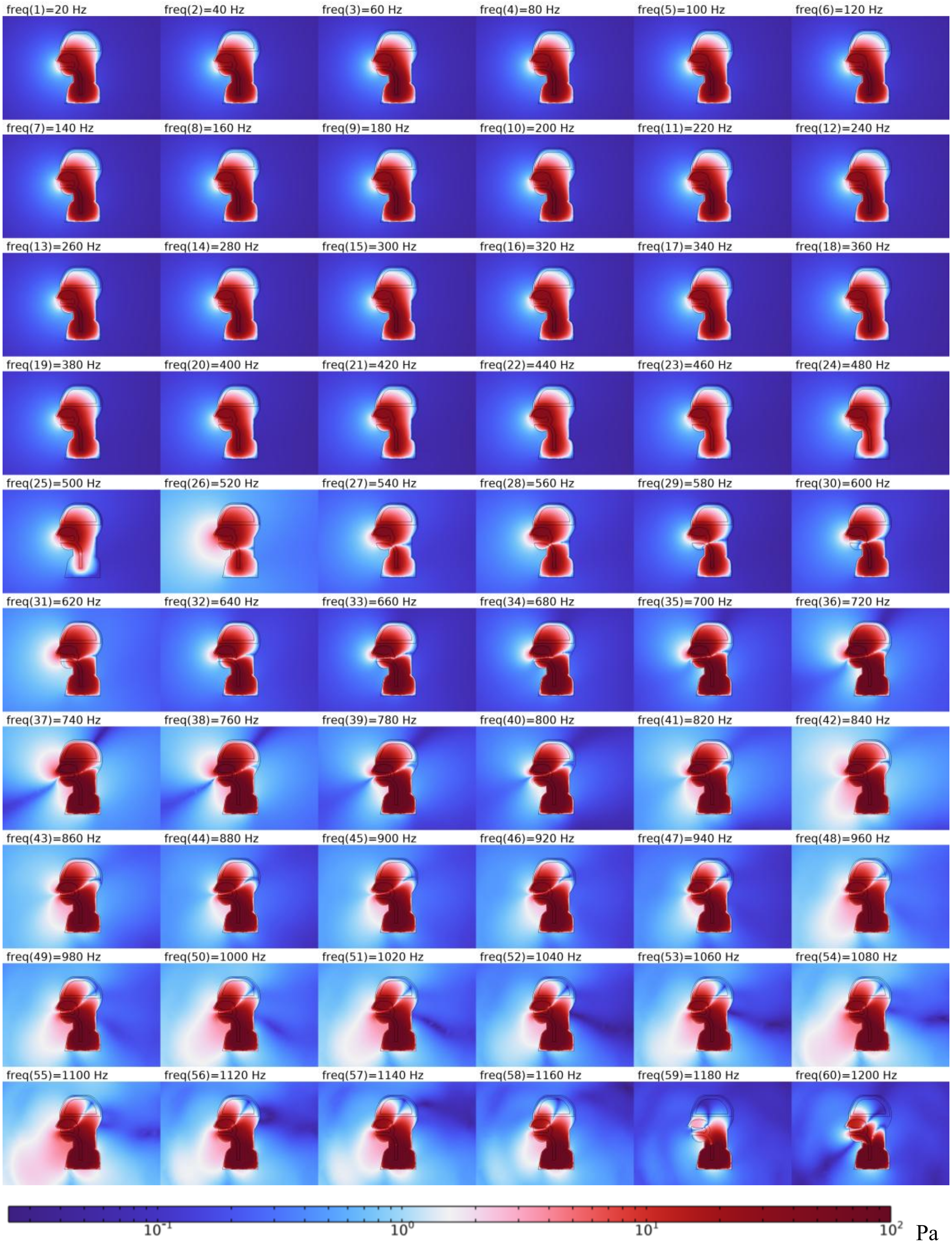

Figure S7: Acoustic pressure distribution in the brain during self-vocalization (singing) across frequencies from 20 Hz to 1200 Hz.

Self-Vocalization (Humming)

Humming, characterized by a closed mouth, shows the highest acoustic pressures among the three conditions. The legend values are significantly higher, reflecting enhanced energy confinement.

- **Low Frequencies (20–200 Hz):** Humming generates very high acoustic pressures at low frequencies, with significant resonance within the enclosed spaces of the nasal and oral cavities. This leads to strong vibrations within the brain, facilitating better mechanical stimulation.
- **Mid Frequencies (200–600 Hz):** The mid-frequency range exhibits pronounced resonance effects, with exceptionally high pressures around 400–500 Hz. The closed-mouth condition leads to stronger standing waves and nodes, indicating very effective constructive interference.
- **High Frequencies (600–1200 Hz):** High-frequency pressures in humming show rapid attenuation, similar to the other conditions.

The analysis of Figures S6, S7, and S8 demonstrates the varying effectiveness of passive listening, singing, and humming in generating brain vibrations across different frequencies. Low frequencies show better penetration and resonance, particularly in self-vocalization scenarios. Mid frequencies around 400–500 Hz exhibit strong resonance effects, while high frequencies are attenuated rapidly. Humming, with its closed-mouth condition, shows the most substantial vibrational impact, yielding the highest modeled intracranial acoustic pressures among the tested conditions. Therapeutic implications remain speculative and require empirical validation.

Conclusion

In conclusion, the FEM simulations provide a quantitative contrast between modeled conditions (self-vocalization vs. passive listening) and suggest that self-initiated vocalization—particularly closed-mouth humming—can plausibly couple more mechanical energy into intracranial tissues than equally loud external sounds under the modeled assumptions. These findings are best interpreted as hypothesis-generating and motivate measurement-based validation. The simulations do not, by themselves, establish any effect on Aβ transport or clearance, and future work should validate cranial vibration/pressure patterns empirically (e.g., with skull-surface accelerometry and cranial phantoms incorporating tissue-mimicking materials and a CSF-like fluid layer) and then test mechanistic endpoints in controlled in vivo and ex vivo models.

Model limitations and validation roadmap.

The current FEM model simplifies anatomy and material properties and does not fully incorporate frequency-dependent damping in living tissues, detailed CSF interfaces, fluid–structure coupling, or physiological pulsatility. To empirically ground the predictions, a practical next step is to (i) measure skull-surface vibration during controlled self-vocalization and passive listening using standardized accelerometer placement (e.g., mastoid and forehead/vertex), and (ii) validate attenuation and spatial patterns in a cranial phantom with tissue-mimicking materials and a fluid layer approximating CSF. These measurements would enable calibration of damping parameters and guide model refinement toward coupled acoustic–solid and fluid–structure simulations.

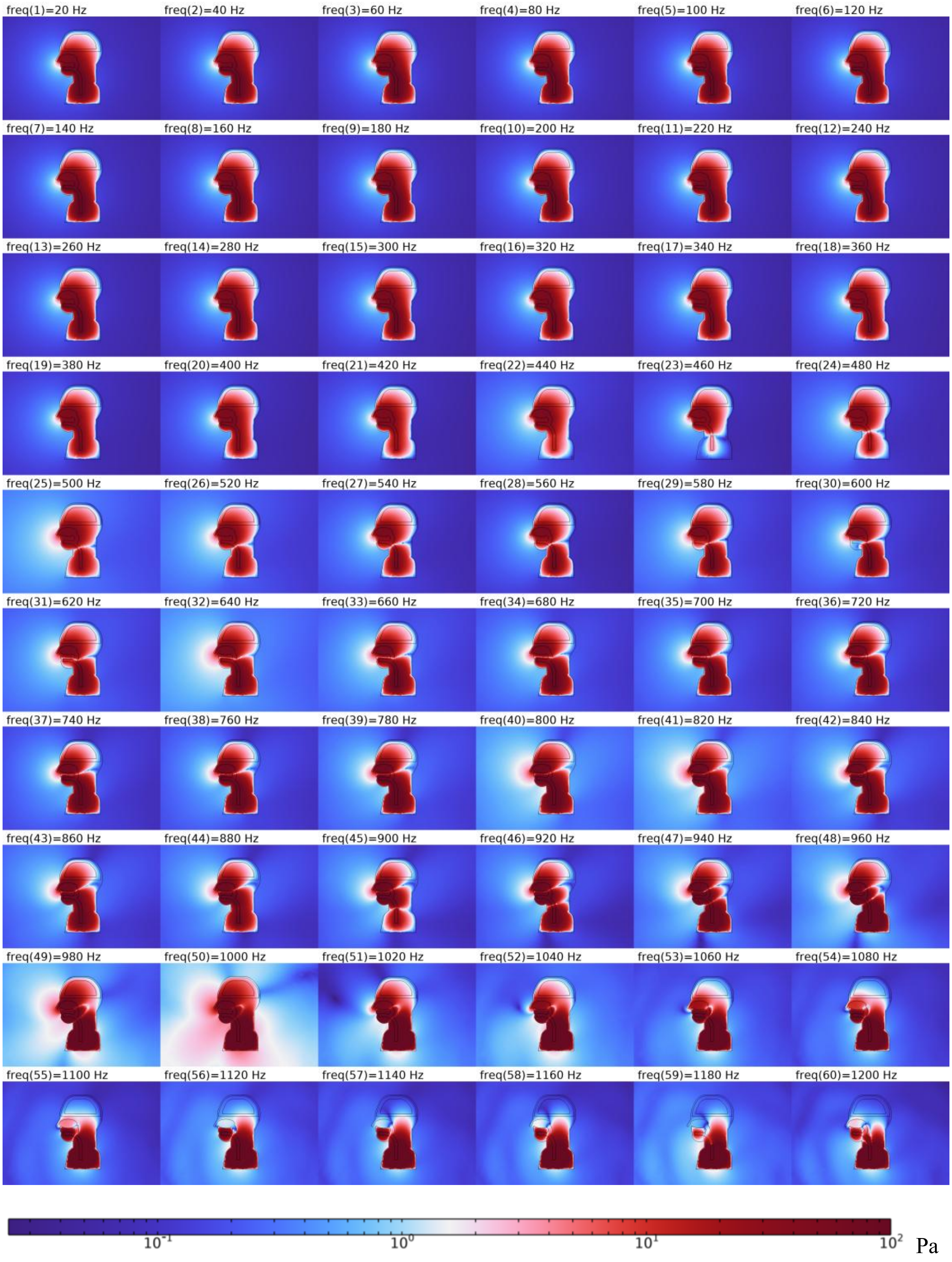

Figure S8: Acoustic pressure distribution in the brain during self-vocalization (humming) across frequencies from 20 Hz to 1200 Hz.
